# An ecological momentary assessment study assessing repetitive negative thinking as a predictor for psychopathology

**DOI:** 10.1371/journal.pone.0318453

**Published:** 2025-03-26

**Authors:** Julia Funk, Celina Müller, Tabea Rosenkranz, Benjamin Aas, Cristina Botella, Felix Burkhardt, Johnny R.J. Fontaine, Mads Frost, Azucena Garcia-Palacios, Christiane Hoessle, Veerle E.I. Huyghe, Johanna Löchner, Alexandra Newbold, Reinhard Pekrun, Belinda Platt, Klaus Scherer, Gerd Schulte-Körne, Björn W. Schuller, Varinka Voigt, Maria Voss, Edward Watkins, Thomas Ehring

**Affiliations:** 1 Department of Psychology, LMU Munich,; 2 Department of Child and Adolescent Psychiatry, Psychosomatics and Psychotherapy, University Hospital, LMU Munich, Germany; 3 Universitat Jaume I, Spain; 4 audEERING, Germany; 5 Department of Work, Organization and Society, Ghent University, Belgium; 6 Monsenso A/S, Copenhagen, Denmark; 7 Department of Child and Adolescent Psychiatry, Psychosomatics and Psychotherapy, University of Tübingen, Germany; 8 German Center for Mental Health (DZPG), partner site Tübingen,; 9 Mood Disorders Centre, University of Exeter,; 10 Department of Psychology, University of Essex,; 11 Institute for Positive Psychology and Education, Australian Catholic University,; 12 University of Geneva,; 13 German Center for Mental Health (DZPG), partner site Munich; Kazimierz Wielki University in Bydgoszcz: Uniwersytet Kazimierza Wielkiego w Bydgoszczy, POLAND

## Abstract

Repetitive negative thinking (RNT), an important transdiagnostic process, is commonly assessed using trait questionnaires. While these instruments ask respondents to estimate their general tendency towards RNT, ecological momentary assessment (EMA) allows to assess how much individuals actually engage in RNT in their daily lives. In a sample of *N* =  1,176 adolescents and young adults, we investigated whether average levels of RNT assessed via EMA predicted psychopathological symptoms. Adjusting for trait RNT measures and baseline scores on outcome measures, we found that average levels of RNT assessed via EMA significantly predicted higher depressive and anxiety symptoms as well as lower mental well-being at baseline, one-, three-, and twelve-month follow-up. Exploratory analyses of the association between temporal dynamics of RNT (e.g., RNT inertia) and psychopathological symptoms yielded inconsistent results. The high predictive power of average scores on the EMA-based RNT measure suggests that EMA is a promising tool for assessing RNT.

## Introduction

Repetitive negative thinking (RNT), a widely studied transdiagnostic process, is a style of thinking focused on negative content and experienced as intrusive and difficult to disengage from [1,2]. It can for example occur in the form of depressive rumination [3] or worrying about the future [4]. Traditionally, researchers have used trait questionnaires to investigate RNT or retrospective questionnaires assessing RNT during a defined interval [e.g., 5,6-8]. Thus, these questionnaires ask respondents to provide an estimate of their general tendency towards RNT or to indicate how much they engaged in RNT over a certain period of time (e.g., past days, weeks, months). Numerous studies have found that patients with mental disorders, such as depression or anxiety disorders, score higher on these trait RNT questionnaires than healthy controls [e.g., 2,9,10]. Additionally, high scores on trait RNT measures have consistently been found to predict the development of future mental health problems [e.g., 11,12-14]. Furthermore, a study on RNT across the life span specifically highlighted the role of RNT in adolescents’ and young adults’ mental health, showing that scores on a trait RNT measure peaked in young adulthood [15].

While studies using trait questionnaires have advanced our understanding of RNT by demonstrating that it is an important factor in the etiology of various mental disorders, measuring RNT via trait questionnaires also has limitations. Importantly, trait measures might by biased by time (retrospective recall) and could reflect metacognitive beliefs about RNT instead of capturing how much a person actually engages in RNT in their daily life [16–19]. A recent review specifically raised concerns about using trait instruments to assess processes such as RNT in adolescents and young adults as cognitions and emotions underly strong temporal fluctuations in these age groups [20].

### Ecological momentary assessment of RNT

In order to overcome limitations of traditional measures and increase ecological validity in the assessment of RNT, recent studies employed ecological momentary assessment (EMA) [21–27]. EMA is an increasingly popular method used to assess psychological processes in (nearly) real time [28,29], for example via participants’ smartphones. In EMA studies measuring RNT, participants are instructed to repeatedly fill out short surveys on how much they currently engage in worry, rumination, or repetitive thinking [24,25]. Notably, EMA studies have typically found only small to moderate correlations between average scores on EMA-based RNT measures and trait questionnaire measures of RNT [22,24,30], indicating that EMA and trait questionnaires assess different aspects of RNT. It seems likely that EMA-based measures might indeed better capture actual levels of RNT in daily life, whereas trait measures that require retrospective recall could more strongly be influenced by metacognitive beliefs about RNT. Moreover, first results suggest that EMA captures features of RNT that may be relevant to the development and maintenance of psychopathology. Specifically, average levels of RNT measured via EMA were found to predict a range of mental health outcomes such as depressive symptoms, anxiety symptoms as well as disordered eating behavior [22–25,31]. As such, EMA could increase the ecological validity in assessing RNT and the associated risk for psychopathology, particularly in adolescents and young adults.

### Value of EMA RNT measures for the prediction of psychopathology

Despite its potential, it is not yet clear how much additional value assessing RNT using EMA holds for the prediction of psychopathology. That is, studies testing whether RNT measured via EMA predicts psychopathological symptoms while adjusting for trait RNT questionnaires are still scarce and inconclusive. One study [22] found that average scores of stress-reactive rumination measured via EMA significantly predicted depressive symptoms when adjusting for trait RNT measures. However, other studies either did not that EMA-based RNT measures predict psychopathological symptoms when adjusting for trait RNT measures [23] or did not control for trait RNT measures in their analyses [24–26]. Similarly, studies investigating whether RNT measured via EMA predicts psychopathological symptoms at a later time point while adjusting for the effect of baseline symptoms have yielded inconsistent results. Some [22,24,32] but not all studies [23,26] found that average scores of RNT measured via EMA predicted psychopathological symptoms when adjusting for baseline symptomatology.

A possible reason for the discrepancies could be that earlier EMA studies have used different measures for momentary RNT. An important distinction between RNT measures (both trait questionnaires and items used for EMA assessment) is whether these are *content-dependent* or *-independent*. Some measures are *content-dependent* in that they assess RNT with respect to (disorder-)specific thought content. Examples are the Ruminative Response Scale [8], which measures the extent to which individuals engage in rumination *about* their own sad mood, or the Penn State Worry Questionnaire [7] assessing worrying *about* the future. In contrast, *content-independent* RNT measures [e.g., the Perserverative Thinking Questionnaire; 5] assess process features of RNT that are shared across different forms of RNT and independent of specific thought content, such as intrusiveness, repetitiveness or uncontrollability of thinking.

Most EMA studies to date have adapted different content-dependent trait RNT measures to assess momentary rumination or worrying in daily life [22,25,26,30]. However, adapting content-independent, i.e., process-focused, RNT measures could also be a promising avenue in EMA research. Content-dependent RNT measures have a narrower focus as they assess specific forms of RNT which characteristically affect individuals with particular types of psychopathology (e.g., worrying as a symptom of generalized anxiety disorder). In comparison, content-independent, process-focused RNT measures might be less confounded with certain symptom domains and can therefore be hypothesized to be more independent and better predictors of different mental health problems. In line with this notion, studies investigating shared and unique aspects of rumination and worrying suggest that shared components reflecting process features of RNT are a better predictor of both depressive and anxiety symptoms than unique ones [14,33–35]. In a recent study, we therefore adapted the Perseverative Thinking Questionnaire (PTQ) – a content-independent process-focused trait RNT measure – to assess momentary RNT in daily life [24]. The new PTQ_EMA_ consists of four items measuring repetitiveness, intrusiveness, and uncontrollability of thinking as well as the distress related to the thinking process and has demonstrated good psychometric properties. In addition, high average scores on this EMA-based RNT measure predicted higher depressive, anxiety and stress symptoms when adjusting for baseline symptoms [24]. The first aim of the current study was to investigate whether the EMA-based RNT measure also predicts psychopathology when adjusting for established trait RNT measures (in addition to adjusting for baseline symptomatology).

### Fluctuations in RNT as a predictor of psychopathology

In addition to providing a better estimation of the frequency and severity of RNT in daily life, EMA provides an excellent means of recording temporal dynamics of RNT. Investigating fluctuations in RNT over time could improve understanding of how RNT leads to a deterioration of mental health. In fact, several theoretical concepts of RNT make assumptions about the temporal dynamics of the process. The habit account of RNT proposes, for example, that RNT initially occurs as a goal-directed attempt to mentally solve problems but then becomes maladaptive by turning into a mental habit that is rigidly triggered in various settings [2,36]. Moreover, RNT has been classified as a dysfunctional emotion regulation strategy that contributes to psychopathology when used in an inflexible and rigid manner [37,38]. Hence, theoretical accounts of RNT assume that RNT is especially maladaptive when inert and resistant to change over time, that is, when a person gets “stuck” in negative thought spirals.

In EMA research, three indices are commonly used to estimate how psychological processes fluctuate over time: *inertia*, *variability,* and *instability* [39–41]. Inertia is formally defined as the first-order autocorrelation of processes assessed repeatedly [42–45]. Thus, high inertia of RNT reflects a high temporal dependency of the repeatedly assessed RNT scores, with scores at each time point being strongly predicted by those assessed at the preceding time point. Variability, on the other hand, is estimated by computing the within-person standard deviation (*SD*) of a process over time [46]. High variability of RNT thus reflects a high magnitude of fluctuations in scores, meaning that a person showed both relatively high and low RNT scores over the measurement period relative to their mean RNT score. While inertia reflects temporal dependency and variability reflects the magnitude of fluctuations, the third index, *instability*, captures both components. Instability can be calculated by computing the root mean squared successive difference (RMSSD) of a time series [40]. High instability of RNT could either reflect high RNT variability, low RNT inertia, or a combination of both.

Prior EMA research has investigated all three indices to test how fluctuations in affect relate to psychopathology. Results point towards complex associations [41], for example, showing that symptoms of mood disorders are associated with high inertia [43–45,47], both high [39,47] and low variability [48], and high instability [49] of (particularly negative) affect. These seemingly contradictory findings may reflect the fact that the different fluctuation parameters describe different components of fluctuations in affect, for example, temporal dependency or magnitude of fluctuations. For example, both high temporal dependency of negative affect on a consistently low level (high inertia and low variability) and steadily increasing negative affect (high inertia and high variability) could be linked to psychopathology. Moreover, the complex picture is in line with theoretical accounts of emotion regulation proposing that both hyper- and insensitive affective reactions to changing contexts can be maladaptive [50].

While prior EMA studies have mainly explored how fluctuations in emotional states relate to mental health, theorical assumptions about temporal dynamics of RNT make it worthwhile to apply similar indices to RNT. Investigating RNT inertia, for example, would enable one to investigate the habit model of RNT according to which RNT is especially dysfunctional when inert and resistant to change, that is, when it has become a mental habit. However, only two studies to date have examined RNT fluctuations and their associations with psychopathology empirically [51,52]. In these studies, high inertia of RNT was indeed positively associated with (residual) depressive symptoms in individuals with current or past depression [51] as well as with sub-clinical depressive symptoms in a healthy sample [52]. Further studies are needed to test whether the findings regarding RNT inertia replicate. In addition, to get a nuanced understanding of which dynamic patterns of RNT can be dysfunctional, it appears promising to investigate all three commonly used fluctuation indices (inertia, variability and instability) based on EMA RNT data within one sample.

### Study aims

EMA promises to increase ecological validity in the assessment of RNT, particularly in young age groups. However, it is not yet clear how much value measuring RNT via EMA provides for the prediction of psychopathology. In the current study, we aimed to investigate whether the new content-independent EMA-based RNT measure (PTQ_EMA_) developed by [24] longitudinally predicts mental health outcomes in a pan-European sample of adolescents and young adults. Specifically, we hypothesized that average scores on the PTQ_EMA_ would predict current and later depressive and generalized anxiety symptoms as well as reduced mental well-being when adjusting for established trait RNT measures and baseline scores on the corresponding outcome measure. Our secondary aim was to explore the link between fluctuations in RNT during the EMA phase and mental health. As there is only a small number of prior studies, none of which has tested different fluctuation indices within the same sample, no a-priori hypotheses were tested. Instead, we investigated associations between RNT dynamics psychopathological symptoms and mental well-being in an exploratory way. We simultaneously tested whether RNT inertia and variability predict depressive symptoms, generalized anxiety symptoms, and mental well-being to differentiate between the impact of temporal dependencies and magnitude of fluctuations in RNT. In separate models, we investigated associations between RNT instability - as a combined index of temporal dependency and variability – and all outcomes.

## Method

### Transparency and openness

The current study was not preregistered. Data, codebook, and analytic code can be found on the Open Science Framework platform (OSF; https://osf.io/dm2ab/). We report all data exclusions, all manipulations, and all measures in the study. This study is a secondary data analysis of data collected within the ECoWeB (emotional competence for well-being in young adults) cohort multiple randomized controlled trial (cmRCT) [53]. Our sample size was determined by the number of participants completing an EMA assessment as part of two parallel randomized controlled trials within the ECoWeB cmRCT. Data was collected online in four countries (Belgium, Germany, Spain, United Kingdom). All study procedures were approved by the Ethics Committees of all trial sites before data collection began (Ethics Committee of the Universitat Jaume I de Castellon, Spain, 14 May 2019, reference number CD/023/2019; Ethics Committee of the LMU Munich, University Hospital, Germany, 4 September 2019, reference number 19-468, 19-315; CLES Psychology Ethics Committee of the University of Exeter, 23 July 2019, reference number eCLESPsy000048 v3.0; Committee for Medical Ethics of the University of Ghent, Belgium, 17 October 2019, reference number 2019-1069). The study procedure of the ECoWeB cmRCT is in accordance with the declaration of Helsinki. All subjects gave written informed consent before participating in the study. For subjects under the age of 18 years, parental written informed consent was collected additionally before study participation. The EcoWeB cmRCT study protocol was registered at ClinicalTrials.gov (Number of identification: NCT04148508). For the purposes of the current analyses, the first and second author accessed the anonymized data set from 20 March 2023 to 10 July 2023, they did not have access to information that could identify individual participants during or after data collection.

### Participants

The sample consisted of adolescents and young adults taking part in the ECoWeB cmRCT [53]. Inclusion criteria were (1) age 16 to 22 years, (2) living in Belgium, Germany, Spain or the UK (3) fluency in at least one of Dutch, English, German or Spanish, (4) written informed consent and written informed parental consent if under 18 years in Germany and Belgium and (5) regular access to an Android or iOS smartphone. Individuals with current or lifetime major depressive disorder, current use of antidepressants or psychological interventions, a history of psychosis, bipolar disorder, substance dependence or other severe psychiatric disorder, or current suicidality were excluded from participation. Participants were recruited via online and website advertising, a social media and press campaign, newsletters and other circulars, and noticeboards within schools, colleges, and universities. As EMA data were available for a subset of *N* =  1,776 participants of the full sample (*N* =  3,794), all statistics reported hereafter pertain to this subset. Table 1 provides an overview of the demographic characteristics of the sample.

**Table 1:** Demographic variables.

Variable		Descriptive statistic
Age		18.87 (1.96)
Gender	female	80.61%
	male	18.28%
	both	0.85%
	neither	0.26%
Ethnicity	white	85.20%
	mixed or multiple ethnicities	5.38%
	Asian	4.68%
	black	1.70%
	Arab or middle eastern	0.06%
	other ethnic group	1.62%
	prefer not to say	0.85%
Highest level of education	elementary or primary school	2.64%
	lower secondary school	31.38%
	upper secondary school or further education college	56.63%
	higher education not at university (e.g., technical college)	4.08%
	undergraduate degree	5.02%
	postgraduate degree	0.26%
Current occupation	students in secondary education	24.49%
	students in university or higher education technical college	41.24%
	working fulltime including caring for dependents (e.g., children)	2.55%
	former students who left or completed secondary school and were not working or studying (yet)	7.65%
	former student who left or completed university or higher education technical college and were not working (yet)	2.47%
	prefer not to say	21.20%

*Note*. Mean (and standard deviation) is reported for age, percentages are reported for each level of the categorical variables.

### Measures

All measures used in the current study were administered in validated versions in either English, Spanish, German or Dutch.

#### Trait rumination.

The 5-item brooding subscale [54] of the Ruminative Response Scale (RRS-B) was used as a measure of trait rumination. In the RRS, respondents are asked to indicate what they generally do when they feel sad, down or depressed. Respondents are instructed to rate items such as “When I feel sad, down or depressed, I think `What am I doing to deserve this?” on a 4-point scale ranging from 1 “almost never” to 4 “almost always”. The RRS-B subscale has demonstrated acceptable internal consistency and associations with current and later depressive symptoms [54]. Cronbach’s alpha in this sample ranged between.68 and.77.

#### Trait worrying.

The Penn State Worry Questionnaire - Abbreviated [PSWQ-A; 55] was administered to assess trait worrying. In this 8-item questionnaire, respondents are instructed to answer items such as “Many situations make me worry” on a scale from 1 “not typical” to 5 “very typical”. The measure has demonstrated high internal consistency, adequate test-retest reliability as well as good convergent and divergent validity [55]. In the current sample, Cronbach’s alpha ranged between.91 and.93.

#### Depressive symptoms.

As a measure of depressive symptoms, the Patient Health Questionnaire-9 [PHQ-9; 56] was administered. The PHQ-9 asks respondents to indicate how much problems such as “little interest or pleasure in doing things” have bothered them in the last two weeks on a scale ranging from 0 “not at all” to 3 “nearly every day”. The PHQ-9 is a widely used and well validated measures of depressive symptoms [56]. Cronbach’s alpha in the current study ranged between.73 and.83

#### Generalized anxiety symptoms.

General anxiety symptoms were assessed using the Generalized Anxiety Disorder-7 questionnaire [GAD-7; 57]. In the GAD-7, respondents are instructed to indicate how often problems such as “feeling nervous, anxious or on edge” have bothered them in the last two weeks on a scale from 0 “not at all” to 3 “nearly every day”. The GAD-7 is a widely used measure for anxiety symptoms that demonstrated good psychometric properties [57]. In the current sample, Cronbach’s alpha ranged between.82 and.86.

#### Mental well-being.

The 14-item Warwick-Edinburgh Mental Well Being Scale [WEMWBS; 58] was administered as a measure for mental well-being. Respondents are asked to rate how much experiences such as “I’ve been feeling optimistic about the future” applied to them in the last two weeks on a scale from 1 “none of the time” to 5 “all of the time”. The WEMWBS is a well-validated scale with good psychometric properties [59]. Cronbach’s alpha in the current sample ranged between.86 and.90.

#### EMA measure of RNT.

To measure RNT in participants’ daily life, we administered our recently developed 4-item Perseverative Thinking Questionnaire _EMA_ [PTQ _EMA_; 24]. The PTQ_EMA_ is based on the Perseverative Thinking Questionnaire [PTQ; 5] and assesses content-independent process features of RNT, i.e., (i) *repetitiveness,* (ii) *intrusiveness*, (iii) *uncontrollability* of thinking as well as (iv) *distress* associated with the thoughts. Participants are instructed to rate the following four items on a seven-point scale ranging from 1 “not at all” to 7 “very much”: (i) “The same negative thoughts keep going through my mind again and again”, (ii) “Negative thoughts come to my mind without me wanting them to”, (iii) “I get stuck on certain negative thoughts and can’t move on” and (iv) “I feel weighed down by negative thoughts”. In a validation study, the PTQ_EMA_ demonstrated excellent between-person reliability and average scores on the measure predicted depression, anxiety, and stress symptoms [24]. In the current study, the PTQ_EMA_ was incorporated into the app used by all trial participants [53]. Over a period of 10 days, participants received 5 beeps a day on their smartphones as prompts to complete the EMA questions. Intervals between the beeps varied randomly in length, however, participants could select a window of hours in which they wanted to receive the beeps throughout the day. The temporal difference between beeps within one day varied between 90 and 120 minutes. The reason for this sampling plan is that a duration of 10 days with a frequency of 5 beeps per day was determined to be the optimal tradeoff between participant burden and information gain in the validation study of the EMA measure [24]. Between-person internal consistency of the measure was high in the current study with Cronbach’s alpha = .95.

### Procedure

A detailed description of the procedure of the underlying trials including all assessed measures can be found in the study protocol [53]. In the following, we will focus on parts of the procedure relevant to the current research question. After having been screened for eligibility, all participants completed the baseline assessment including measures of trait rumination, trait worrying, depressive and anxiety symptoms as well as mental well-being. Consequently, participants were randomly allocated to either (i) use a self-monitoring app, (ii) to additionally receive generic cognitive-behavioral therapy self-help via app, or (iii) to additionally receive personalized emotional competence training self-help via app. Importantly, each condition included a self-monitoring option in the app. In each condition, participants were automatically enabled and instructed to download the app on their smartphone. The app was designed to be used for a period of three months. The 10-day EMA assessment took part from Day 5 to Day 14 of the app usage. One-, three- and twelve-months post-randomization, participants completed follow-up assessments comprising the same measures as the baseline assessment. Within our study sample with EMA data (*N* =  1,776), 941 participants completed the one-month follow-up, 880 completed the three-month follow-up and 800 completed the twelve-month follow-up.

### Data Analysis

All analyses were conducted in R [R version 4.0.3; 60].

#### The average score on the PTQ_EMA_ as a predictor of depressive symptoms, generalized anxiety symptoms, and mental well-being.

We conducted linear regression analyses to investigate whether average scores of the PTQ_EMA_ predicted sum scores on the PHQ-9, GAD-7 and WEMWBS at baseline and follow-ups. Average scores on the PTQ_EMA_ measure were computed by calculating the mean sum score of the four EMA RNT items across all completed measurement time points for each participant. In each of the regression models, we controlled for sum scores on the trait RNT measures, i.e., the RRS-B and the PSWQ-A. In models predicting outcomes at follow-up, we additionally controlled for the baseline score on the respective outcome measure (i.e., PHQ-9, GAD-7 or WEMWBS, respectively) and the effects of trial condition. List-wise deletion was used to deal with missing data in the outcome variables. To account for the fact that there were three dependent variables at each time point, we applied Bonferroni-correction for multiple comparisons to the alpha level (corrected *α* =  0.017).

#### RNT dynamics as predictors of depressive symptoms, generalized anxiety symptoms, and mental well-being.

RNT inertia was calculated by computing autoregressive coefficients point according to Trull et al., 2015, indicating how well sum scores on the PTQ_EMA_ at each time point are predicted by scores at the preceding time. RNT variability was calculated by computing the participant-specific standard deviation from the participant-specific mean sum score on the PTQ_EMA_. Consequently, we conducted linear regression analyses to test whether inertia and variability predicted sum scores on the PHQ-9, GAD-7 and WEMWBS at baseline and follow-ups. We controlled for the same variables as in the models testing the average scores on the PTQ_EMA_ as a predictor and additionally controlled for average scores on the PTQ_EMA_. RNT instability was calculated by computing the root mean square of successive differences (RMSSD) in sum scores on the PTQ_EMA_ for each participant according to [40]. As for inertia and variability, we conducted linear regression analyses adjusting for the same variables to test whether RNT instability predicted sum scores on the PHQ-9, GAD-7 and WEMWBS at baseline and follow-ups. Due to missing EMA data, RNT inertia and instability could only be calculated for a subset of 665 and 994 participants, respectively. A Bonferroni-corrected alpha level was used for significance testing (corrected *α* =  0.017).

## Results

### Data cleaning and compliance

From Day 5 until Day 14 of their app usage, participants should have received 50 push-notifications (beeps) to answer the EMA questions. However, due to a fire in the server center and subsequent app outage for a month, participants received a varying number of beeps (e.g., the EMA phase started later than planned for some participants, EMA questions were sent either more or less often than planned). To maximize our sample size and analyze the EMA data despite these technical issues, we preprocessed the data in four steps. First, we split EMA data from an extended window (Day 1 until Day 60 of the app usage) into blocks where participants received consecutive beeps (less than two days difference between two beeps). Then, we filtered out blocks where participants received between 50 and 70 beeps as only a minority of participants (*n* =  70) had blocks of exactly 50 consecutive beeps. In a third step, for participants with more than one block of 50 to 70 consecutive beeps, we filtered out the block with the highest answer rate. Finally, all participants who did not respond to the EMA assessment at all in the identified window were removed from the data set. All statistics reported in the paper pertain to the data set resulting from this data cleaning procedure. We additionally performed a sensitivity analysis on a subset of *N* =  796 participants in which we only included EMA data from Day 5 until day 14 of the app usage (planned EMA period). The sensitivity analysis did not substantially differ from the primary analysis regarding results on average scores on the EMA-based RNT measure. However, results differed in terms of results on the dynamic parameters, potentially due to decreased power in the more complex models. The analytic code used for the sensitivity analysis can be found on OSF (https://osf.io/dm2ab/).

### Answer rate EMA assessment

The mean answer rate for the EMA assessment was 26% (*SD* = 26%).

### Correlations between the PTQ_EMA_ and the trait RNT measures

Table 2 shows means, standard deviations and Pearson correlation coefficients for the three RNT measures, RRS-B (sum score at baseline), PSWQ-A (sum score at baseline) and average sum scores on the PTQ_EMA_. All correlations between RNT measures were significant. The correlation between RRS-B and PSWQ-A can be classified as large whereas the correlations between the two trait measures and the PTQ_EMA_ can be classified as moderate [61].

**Table 2:** Descriptive statistics and Pearson correlations for the RNT measures.

	*Mean*	*SD*	PTQ_EMA_(mean)	RRS-B	PSWQ-A
PTQ_EMA_(mean)	9.50	4.92	1	.35	.39
RRS-B	9.86	2.89	.35	1	.51
PSWQ-A	21.77	7.67	.39	.51	1

*Note*. *SD* =  standard deviation; PTQ_EMA_(mean) =  Average sum score on the PTQ_EMA_ across all completed measurement timepoints; RRS-B =  Sum score on the RRS-B at baseline; PSWQ-A =  sum score on the PSWQ-A at baseline; correlations between all RNT measures were significant with p < .001

### The average score on the PTQ_EMA_ as a predictor of depressive symptoms, generalized anxiety symptoms, and mental well-being

#### Depressive symptoms.

Table 3 provides the results for the linear regressions testing the average score on the EMA-based RNT measure as a predictor of depressive symptoms at baseline and at all three follow-ups. Average scores on the PTQ_EMA_ significantly predicted sum scores on the PHQ at baseline when adjusting for trait RNT measures (PSWQ-A and RRS-B sum scores at baseline). They also significantly predicted depressive symptoms at the three follow-up assessments when additionally adjusting for baseline sum scores on the PHQ-9 and trial condition. Only the PTQ_EMA_, but not the two trait RNT measures, significantly predicted depressive symptoms at the twelve-month follow-up.

**Table 3:** Linear regressions predicting depressive symptoms (sum score on the PHQ – 9).

	Baseline				One-month Follow-up			Three-month Follow-up			Twelve-month Follow-up		
*Predictors*	*B (CI)*		*β*	*p*	*B (CI)*	*β*	*p*	*B (CI)*	*β*	*p*	*B (CI)*	*β*	*p*
PTQ_EMA_ (*mean*)	0.12 (0.09 – 0.16)		0.17	**<0.001**	0.20 (0.15 – 0.25)	0.24	**<0.001**	0.21 (0.15 – 0.27)	0.23	**<0.001**	0.13 (0.06 – 0.20)	0.13	**<0.001**
RRS-B at baseline	0.33 (0.26 – 0.40)		0.26	**<0.001**	0.10 (0.02 – 0.19)	0.07	0.022	0.15 (0.05 – 0.26)	0.10	**0.005**	0.08 (-0.05 – 0.20)	0.05	0.244
PSWQ-A at baseline	0.13 (0.10 – 0.16)		0.28	**<0.001**	0.01 (-0.02 – 0.05)	0.02	0.459	-0.02 (-0.06 – 0.02)	-0.03	0.360	0.01 (-0.04 – 0.06)	0.02	0.612
PHQ-9 at baseline					0.44 (0.37 – 0.52)	0.38	**<0.001**	0.42 (0.34 – 0.51)	0.34	**<0.001**	0.38 (0.28 – 0.48	0.29	**<0.001**
Condition (self-monitoring)					0.35 (-0.18 – 0.88)	0.09	0.195	0.42 (-0.20 – 1.04)	0.10	0.185	0.15 (-0.60 – 0.89)	0.03	0.699
Condition (self-monitoring ^+^ EC)					0.27 (-0.25 – 0.78)	0.07	0.314	0.43 (-0.17 – 1.03)	0.10	0.164	0.05 (-0.66 – 0.77)	0.01	0.884
Observations	1176				941			880			800		
*R*^*2*^ / *R*^*2*^ adjusted	0.316/ 0.314				0.325/ 0.320			0.263/ 0.258			0.157/ 0.151		

*Note. B* (CI) = unstandardized regression coefficient (with 95% confidence interval), *β* =  standardized regression coefficient, *p* =  raw *p*-value; bold *p*-values denote significance below *α* =  0.017 (Bonferroni-corrected for multiple dependent variables); *R² (adjusted)* =  (adjusted) coefficient of determination;, self-monitoring =  self-monitoring only app, self-monitoring +  EC =  self-monitoring +  personalized emotional competence training self-help via app. Reference group for condition is self-monitoring +  generic cognitive-behavioral therapy self-help via app.

#### Generalized anxiety symptoms.

Table 4 shows the results of the linear regressions predicting anxiety symptoms at baseline and at all three follow-up assessments. Average scores on the PTQ_EMA_ significantly predicted sum scores on the GAD-7 at all four time points when adjusting for trait RNT measures and additionally adjusting for trial condition and baselines scores on the GAD-7 in the models predicting symptoms at follow-up.

**Table 4:** Linear regressions predicting generalized anxiety symptoms (sum score on the GAD – 7).

	Baseline				One-month Follow-up			Three-month Follow-up			Twelve-month Follow-up		
*Predictors*	*B (CI)*		*β*	*p*	*B (CI)*	*β*	*p*	*B (CI)*	*β*	*p*	*B (CI)*	*β*	*p*
PTQ_EMA_ (*mean*)	0.13 (0.10 – 0.17)		0.17	**<0.001**	0.21 (0.16 – 0.25)	0.25	**<0.001**	0.19 (0.14 – 0.25)	0.22	**<0.001**	0.12 (0.05 – 0.18)	0.13	**<0.001**
RRS-B at baseline	0.27 (0.20 – 0.34)		0.20	**<0.001**	0.07 (-0.01 – 0.15)	0.05	0.068	0.08 (-0.02 – 0.19)	0.06	0.106	0.06 (-0.05 – 0.18)	0.04	0.287
PSWQ-A at baseline	0.23 (0.20 – 0.25)		0.46	**<0.001**	0.10 (0.06 – 0.13)	0.19	**<0.001**	0.07 (0.02 – 0.11)	0.12	**0.002**	0.05 (0.01 – 0.10)	0.10	0.031
GAD-7 at baseline					0.36 (0.29 – 0.42)	0.34	**<0.001**	0.32 (0.24 – 0.41)	0.28	**<0.001**	0.27 (0.17 – 0.37)	0.24	**<0.001**
Condition (self-monitoring)					-0.14 (-0.60 – 0.33)	-0.03	0.572	-0.05 (-0.65 – 0.55)	-0.01	0.879	0.04 (-0.64 – 0.71)	0.01	0.919
Condition (self- Monitoring + EC)					-0.10 (-0.56 – 0.35)	-0.03	0.663	0.07 (-0.51 – 0.65)	0.02	0.810	0.13 (-0.52 – 0.78)	0.03	0.700
Observations	1176				942			886			800		
*R*^*2*^ / *R*^*2*^ adjusted	0.462/ 0.461				0.445/ 0.441			0.279/ 0.275			0.171/ 0.165		

*Note. B* (CI) = unstandardized regression coefficient (with 95% confidence interval), *β* =  standardized regression coefficient, *p* =  raw *p*-value; bold *p*-values denote significance below *α* =  0.017 (Bonferroni-corrected for multiple dependent variables); *R² (adjusted)* =  (adjusted) coefficient of determination;, self-monitoring =  self-monitoring only app, self-monitoring +  EC =  self-monitoring +  personalized emotional competence training self-help via app. Reference group for condition is self-monitoring +  generic cognitive-behavioral therapy self-help via app.

#### Mental Well-being.

Results of the linear regressions predicting well-being at baseline and at the three follow-up assessments are shown in Table 5. Average scores on the PTQ_EMA_ showed significant negative associations with well-being at all four time points. At all three follow-up time points, the average score on the PTQ_EMA_ but not the sum scores on the trait RNT measures significantly predicted sum scores on the WEMWBS when adjusting for baseline scores on the WEMWBS.

**Table 5:** Linear regressions predicting mental well-being (sum score on the WEMWBS).

	Baseline				One-month Follow-up			Three-month Follow-up			Twelve-month Follow-up		
*Predictors*	*B (CI)*		*β*	*p*	*B (CI)*	*β*	*p*	*B (CI)*	*β*	*p*	*B (CI)*	*β*	*p*
PTQ_EMA_ (*mean*)	-0.20 (-0.28 – -0.12)		-0.14	**<0.001**	-0.23 (-0.31 – -0.14)	-0.14	**<0.001**	-0.36 (-0.46 – -0.25)	-0.21	**<0.001**	-0.20 (-0.32 – -0.08)	-0.12	**0.001**
RRS-B at baseline	-0.53 (-0.68 – -0.39)		-0.22	**<0.001**	-0.01 (-0.16 – 0.13)	-0.01	0.853	-0.05 (-0.25 – 0.14)	-0.02	0.578	-0.10 (-0.32 – 0.11)	-0.04	0.338
PSWQ-A at baseline	-0.25 (-0.30 – -0.19)		-0.27	**<0.001**	-0.01 (-0.07 – 0.05)	-0.01	0.682	0.03 (-0.04 – 0.10)	0.03	0.390	-0.02 (-0.10 – 0.07)	-0.02	0.673
WEMWBS at baseline					0.63 (0.57 – 0.69)	0.59	**<0.001**	0.50 (0.42 – 0.57)	0.44	**<0.001**	0.39 (0.30 – 0.47)	0.34	**<0.001**
Condition (self-monitoring)					-0.68 (-1.58 – 0.21)	-0.09	0.134	0.00 (-1.12 – 1.12)	0.00	1.000	-0.26 (-1.52 – 0.99)	-0.03	0.683
Condition (self- Monitoring + EC)					0.32 (-0.55 – 1.19)	0.04	0.475	-0.23 (-1.31 – 0.86)	-0.03	0.682	0.01 (-1.20 – 1.21)	0.00	0.988
Observations	1176				944			885			801		
*R*^*2*^ / *R*^*2*^ adjusted	0.250/ 0.248				0.440/ 0.437			0.288/ 0.283			0.181/ 0.175		

*Note. B* (CI) = unstandardized regression coefficient (with 95% confidence interval), *β* =  standardized regression coefficient, *p* =  raw *p*-value; bold *p*-values denote significance below *α* =  0.017 (Bonferroni-corrected for multiple dependent variables); *R² (adjusted)* =  (adjusted) coefficient of determination;, self-monitoring =  self-monitoring only app, self-monitoring +  EC =  self-monitoring +  personalized emotional competence training self-help via app. Reference group for condition is self-monitoring +  generic cognitive-behavioral therapy self-help via app.

### RNT Dynamics as Predictors of Depressive Symptoms, Generalized Anxiety Symptoms, and Mental Well-Being

#### RNT inertia and variability.

RNT inertia during the EMA phase predicted depressive symptoms at the one-month follow-up. In addition, RNT variability predicted anxiety symptoms at the three-month follow-ups (see Supplementary Material, Tables S1 – S3).

#### RNT instability.

RNT instability negatively predicted depressive symptoms at baseline. No other significant associations of RNT instability with the outcomes were found (see Supplementary Material, Tables S4 – S6).

## Discussion

The current study aimed to investigate whether RNT measured in daily life via EMA predicts psychopathological symptoms and mental well-being. As hypothesized, average scores on the PTQ_EMA_ significantly predicted higher depressive and generalized anxiety symptoms and lower mental well-being at baseline, one-month, three-months, and twelve-months follow-up. Notably, the average score on the PTQ_EMA_ was a more consistent predictor of the mental health outcomes than scores on the trait RNT measures. For example, depressive symptoms at the twelve-month and one-month follow-up and mental well-being at all follow-ups were predicted by average RNT scores on the PTQ_EMA,_ whereas trait RNT measures did not emerge as significant predictors. While several prior studies found that average scores on EMA RNT measures predicted psychopathological symptoms [22–25,31], the present study is one of the first studies to show that this is still true when adjusting for baseline symptoms and trait RNT measures.

When interpreting the results, it should be considered that PTQ_EMA_ is process-focused and content-independent, whereas the trait RNT questionnaires used are content-dependent [RRS-B assessing rumination about one’s own negative mood; 54, PSWQ-A measuring worrying about the future; 55]. This presents a limitation as it could be that our findings are based on a difference between process- vs. content-based measures, regardless of how they are administered. Reassuringly however, [62] used the PTQ_EMA_ in a prospective study that additionally included the PTQ as a content-independent trait RNT measure. Results were similar to the findings of the present study, confirming that assessing content-independent process features of RNT via EMA is indeed a promising method.

In addition to testing the predictive power of average scores on the PTQ_EMA_ measure, we explored whether patterns in the temporal dynamics of RNT across the EMA phase were predictive of mental health outcomes. In sum, results were much less consistent than for average scores on the PTQ_EMA_. Yet, specific associations between certain RNT dynamics and some of the outcomes emerged. High RNT inertia predicted higher depressive symptoms at one-month-follow up, whereas high RNT variability predicted higher generalized anxiety symptoms at three-month follow-up. In addition, high RNT instability, which can reflect low temporal dependency and/or a high amplitude of fluctuations in the repeatedly assessed RNT scores, predicted lower depressive symptoms at baseline but none of the other outcomes. The findings regarding RNT inertia suggest that - in addition to how much individuals engage in RNT on average - an inert pattern of getting stuck in RNT might be linked to the development of depression. In contrast, RNT inertia may be a less important dynamic in anxiety. Generalized anxiety symptoms rather seemed to be linked to high variability, that is individuals showing both relatively high and low RNT scores relative to their average RNT score.

Even though we found these associations, the findings should be interpreted with great caution as the analyses were exploratory, and the associations did not emerge consistently across all measurement time points. The inconsistent findings are somewhat paralleled by EMA research on links between *emotion* dynamics and mental health, showing that low well-being and high psychopathological symptoms can be linked to high inertia [41,43–45,47], both high [39,41,47] and low variability [48] and high instability [41,49] of affect. It has been argued that healthy emotional functioning might be characterized by flexible emotional changes (low inertia) within a moderate range (low variability/instability) [41]. Similar reasoning could also be applied the process of RNT, suggesting that both rigidly engaging in RNT and getting stuck in negative thoughts over a longer period of time as well as engaging in RNT with substantially higher intensity than usual from time to time could be maladaptive and result in depression or anxiety, respectively. However, these ideas remain speculative at this stage and should be investigated more systematically in future research.

### Limitations

We administered the PTQ_EMA_ in a non-clinical sample of young adults who did not meet the criteria for a mental disorder at the beginning of the study. As a result, we only investigated associations between scores on the PTQ_EMA_ and subclinical symptoms, which limits the generalizability of findings to clinical populations. A next step for future studies should be to examine whether findings replicate when the measure is administered in a sample of patients with current diagnoses of or heightened risk for mental disorders. As RNT is considered to be a transdiagnostic factor, studies in samples including a broader spectrum of mental disorders beyond depression and anxiety disorder would be especially informative. Relatedly, the exclusion of participants with high symptom scores may have reduced the variance of symptom levels or RNT. A second limitation of the current study is that the response rate within the EMA assessment was low, with participants answering on average 26% of the EMA questions. There is a number of reasons that potentially contributed to the low response rate. In the current study, EMA was administered as part of self-help apps for training emotional competencies and promoting mental health [53]. The usage of stand-alone self-help apps in general is characterized by low compliance and high rates of drop out [63]. Additionally, participants might have been more motivated to use other app contents instead of completing the EMA questions. Considering that our sample consisted of adolescents and young adults, an age group, where achieving good compliance with EMA is challenging [64,65], the relatively high sampling frequency (5 beeps per day) might have been too ambitious. Moreover, severe technical errors due to an outage in the server center during the EMA phase (see section Data cleaning and Compliance) likely influenced the response rate. The low response rate should be considered when interpreting the findings of the current study. The fact that average scores on the PTQ_EMA_ predicted psychopathology even with this low response rate can be interpreted in favor of the robustness of this measure. At the same time, it is conceivable that the low response rate in our study may have reduced the predictive power of the indices for RNT dynamics. As the computation of both the inertia and the instability coefficient relies on consecutive data points, it is likely that these indices were affected by the substantial amount missing data. To obtain reliable results with regards to RNT dynamics, future studies on the topic should take measures to ensure a sufficiently high response rate. Relatedly, the low compliance rates in the current study prevented us from testing more sophisticated indices of RNT dynamics. Future research should aim to more systematically disentangle different components of RNT dynamics, including short-term and long-term changes, as well as interactions between average levels of RNT and RNT inertia or variability.

## Conclusions and outlook

Our findings showed that measuring RNT in daily life using EMA provides additional value for the prediction of psychopathology and mental well-being in adolescents and young adults. Importantly, average scores on the EMA measure predicted mental health outcomes more consistently than established trait RNT questionnaires. Thus, it appears promising to include EMA measures in studies investigating RNT, particularly when research is conducted in adolescent and young adult samples. Measuring RNT via EMA could for example be useful for assessing the effects of interventions that are designed to reduce RNT [66]. In the current study we also investigated how different patterns in the temporal dynamics of RNT are related to mental health outcomes. However, as results were inconclusive, more research is needed to clarify how stable and dynamic features of RNT in daily life are linked to psychopathology. Further perusing this line of research may not only advance understanding of the mechanisms by which RNT leads to a deterioration of mental health but could also have implications for optimizing interventions. A recent study demonstrated that treatment outcomes can be predicted by certain dynamics in daily symptom profiles during psychotherapy [67]. Similarly, investigating dynamics of daily life RNT during treatments could have potential for improving the prediction of treatment responses and may ultimately facilitate personalization of treatments.

## Supporting information

Supplementary Material Tables The supporting information is included in the file “Supplementary Material RNT in daily life”. (DOCX)
