# Supplementary material for: An ecological momentary assessment study assessing repetitive negative thinking as a predictor for psychopathology
Source: PLoS One. 2025 Mar 26;20(3):e0318453. doi: 10.1371/journal.pone.0318453 (PMC11940665; doi:10.1371/journal.pone.0318453)
Supplement: Supplementary Material Tables — (DOCX) [file pone.0318453.s001.docx]

**Supplementary Material**

Tables S1-3 shows the results of linear regressions testing the effects of inertia and variability of repetitive negative thinking (RNT) on depressive symptoms, generalized anxiety symptoms and mental well-being, respectively. Tables S4-6 show the results of linear regressions testing the effects of RNT instability on the same outcomes.

**Table S1**

|  | **Baseline** | | | | **One-month Follow-up** | | | **Three-month Follow-up** | | | **Twelve-month Follow-up** | | |
| --- | --- | --- | --- | --- | --- | --- | --- | --- | --- | --- | --- | --- | --- |
| *Predictors* | *B (CI)* | | *β* | *p* | *B (CI)* | *β* | *p* | *B (CI)* | *β* | *p* | *B (CI)* | *β* | *p* |
| PTQ_EMA_ (inertia) | 1.36  (-0.16 – 2.88) | | 0.06 | 0.080 | 2.59  (0.80 – 4.39) | 0.11 | **0.005** | 1.16  (-0.99 – 3.30) | 0.05 | 0.291 | 2.13  (-0.41 – 4.67) | 0.08 | 0.100 |
| PTQ_EMA_ (variability) | -0.01  (-0.14 – 0.13) | | -0.01 | 0.896 | 0.10  (-0.06 – 0.26) | 0.05 | 0.224 | 0.14  (-0.06 – 0.34) | 0.07 | 0.165 | 0.12  (-0.11 – 0.36) | 0.05 | 0.312 |
| PTQ_EMA_ (mean) | 0.11  (0.04 – 0.19) | | 0.14 | **0.003** | 0.23  (0.14 – 0.31) | 0.24 | **<0.001** | 0.21  (0.10 – 0.32) | 0.20 | **<0.001** | 0.11  (-0.02 – 0.24) | 0.09 | 0.107 |
| RRS-B at baseline | 0.27  (0.18 – 0.36) | | 0.22 | **<0.001** | 0.12  (0.01 – 0.23) | 0.09 | 0.033 | 0.20  (0.06 – 0.33) | 0.13 | **0.004** | 0.09  (-0.07 – 0.25) | 0.06 | 0.266 |
| PSWQ-A at baseline | 0.15  (0.11 – 0.18) | | 0.33 | **<0.001** | 0.02  (-0.03 – 0.06) | 0.03 | 0.463 | -0.06  (-0.11 – -0.00) | -0.10 | 0.039 | -0.00  (-0.07 – 0.06) | -0.00 | 0.943 |
| PHQ-9 at baseline |  | |  |  | 0.38  (0.29 – 0.47) | 0.33 | **<0.001** | 0.37  (0.26 – 0.48) | 0.29 | **<0.001** | 0.42  (0.28 – 0.55) | 0.30 | **<0.001** |
| Condition (self-monitoring) |  |  |  |  | 0.35  (-0.31 – 1.02) | 0.09 | 0.297 | 0.26  (-0.56 – 1.07) | 0.06 | 0.537 | -0.42  (-1.40 – 0.55) | -0.09 | 0.395 |
| Condition (self-  Monitoring + EC) |  |  |  |  | 0.13  (-0.49 – 0.76) | 0.03 | 0.672 | -0.06  (-0.82 – 0.71) | -0.01 | 0.886 | -0.56  (-1.46 – 0.34) | -0.12 | 0.223 |
| Observations | 665 | | | | 573 | | | 554 | | | 501 | | |
| *R^2^* / *R^2^* adjusted | 0.324 / 0.319 | | | | 0.352 / 0.343 | | | 0.231 / 0.219 | | | 0.174 / 0.160 | | |

*Linear regressions testing the effects of RNT inertia and variability on depressive symptoms (sum score on the PHQ-9)*

*Note. B* (CI)= unstandardized regression coefficient (with 95% confidence interval), *β* = standardized regression coefficient, *p* = raw *p*-value; bold *p*-values denote significance below *α* = 0.017 (Bonferroni-corrected for multiple dependent variables); *R² (adjusted)* = (adjusted) coefficient of determination; , self-monitoring = self-monitoring only app, self-monitoring + EC = self-monitoring + personalized emotional competence training self-help via app. Reference group for condition is self-monitoring + generic cognitive-behavioral therapy self-help via app.

**Table S2**

|  | **Baseline** | | | **One-month Follow-up** | | | **Three-month Follow-up** | | | **Twelve-month Follow-up** | | |
| --- | --- | --- | --- | --- | --- | --- | --- | --- | --- | --- | --- | --- |
| *Predictors* | *B (CI)* | *β* | *p* | *B (CI)* | *β* | *p* | *B (CI)* | *β* | *p* | *B (CI)* | *β* | *p* |
| PTQ_EMA_ (inertia) | -1.02  (-2.42 – 0.38) | -0.04 | 0.154 | 0.75  (-0.80 – 2.29) | 0.03 | 0.343 | -0.56  (-2.53 – 1.42) | -0.02 | 0.580 | 0.81  (-1.53 – 3.14) | 0.03 | 0.499 |
| PTQ_EMA_ (variability) | -0.11  (-0.23 – 0.01) | -0.06 | 0.084 | 0.15  (0.01 – 0.29) | 0.08 | 0.042 | 0.25  (0.06 – 0.43) | 0.12 | **0.009** | 0.04  (-0.18 – 0.25) | 0.02 | 0.747 |
| PTQ_EMA_ (mean) | 0.23  (0.16 – 0.29) | 0.25 | **<0.001** | 0.22  (0.14 – 0.30) | 0.23 | **<0.001** | 0.22  (0.11 – 0.32) | 0.21 | **<0.001** | 0.08  (-0.04 – 0.21) | 0.08 | 0.201 |
| RRS-B at baseline | 0.23  (0.15 – 0.31) | 0.18 | **<0.001** | -0.04  (-0.13 – 0.06) | -0.03 | 0.438 | 0.09  (-0.03 – 0.22) | 0.06 | 0.143 | 0.06  (-0.09 – 0.21) | 0.04 | 0.414 |
| PSWQ-A at baseline | 0.24  (0.21 – 0.27) | 0.49 | **<0.001** | 0.11  (0.07 – 0.15) | 0.23 | **<0.001** | 0.05  (-0.01 – 0.10) | 0.09 | 0.087 | 0.06  (0.00 – 0.13) | 0.12 | 0.046 |
| GAD-7 at baseline |  |  |  | 0.38  (0.29 – 0.46) | 0.37 | **<0.001** | 0.31  (0.19 – 0.42) | 0.27 | **<0.001** | 0.31  (0.18 – 0.44) | 0.27 | **<0.001** |
| Condition (self-monitoring) |  |  |  | -0.12  (-0.69 – 0.46) | -0.03 | 0.687 | -0.66  (-1.41 – 0.09) | -0.16 | 0.083 | -0.57  (-1.47 – 0.33) | -0.13 | 0.215 |
| Condition (self-  Monitoring + EC) |  |  |  | -0.35  (-0.89 – 0.18) | -0.09 | 0.193 | -0.41  (-1.10 – 0.29) | -0.10 | 0.252 | -0.35  (-1.18 – 0.47) | -0.08 | 0.402 |
| Observations | 665 | | | 575 | | | 554 | | | 501 | | |
| *R^2^* / *R^2^* adjusted | 0.506 / 0.502 | | | 0.507 / 0.500 | | | 0.316 / 0.306 | | | 0.191 / 0.177 | | |

*Linear regressions testing the effects of RNT inertia and variability on generalized anxiety symptoms (sum score on the GAD-7)*

*Note. B* (CI)= unstandardized regression coefficient (with 95% confidence interval), *β* = standardized regression coefficient, *p* = raw *p*-value; bold *p*-values denote significance below *α* = 0.017 (Bonferroni-corrected for multiple dependent variables); *R² (adjusted)* = (adjusted) coefficient of determination; , self-monitoring = self-monitoring only app, self-monitoring + EC = self-monitoring + personalized emotional competence training self-help via app. Reference group for condition is self-monitoring + generic cognitive-behavioral therapy self-help via app.

**Table S3**

|  | **Baseline** | | | **One-month Follow-up** | | | **Three-month Follow-up** | | | **Twelve-month Follow-up** | | |
| --- | --- | --- | --- | --- | --- | --- | --- | --- | --- | --- | --- | --- |
| *Predictors* | *B (CI)* | *β* | *p* | *B (CI)* | *β* | *p* | *B (CI)* | *β* | *p* | *B (CI)* | *β* | *p* |
| PTQ_EMA_ (inertia) | -1.07  (-4.22 – 2.07) | -0.02 | 0.503 | -3.44  (-6.47 – -0.40) | -0.08 | 0.026 | -0.37  (-4.19 – 3.45) | -0.01 | 0.850 | -2.79  (-7.22 – 1.63) | -0.06 | 0.216 |
| PTQ_EMA_ (variability) | 0.20  (-0.08 – 0.48) | 0.06 | 0.162 | -0.14  (-0.42 – 0.14) | -0.04 | 0.330 | 0.09  (-0.26 – 0.45) | 0.02 | 0.615 | 0.18  (-0.23 – 0.59) | 0.04 | 0.400 |
| PTQ_EMA_ (mean) | -0.30  (-0.45 – -0.15) | -0.18 | **<0.001** | -0.21  (-0.36 – -0.06) | -0.12 | **0.007** | -0.45  (-0.65 – -0.25) | -0.23 | **<0.001** | -0.20  (-0.44 – 0.03) | -0.10 | 0.086 |
| RRS-B at baseline | -0.62  (-0.81 – -0.43) | -0.26 | **<0.001** | 0.10  (-0.08 – 0.29) | 0.04 | 0.285 | -0.08  (-0.33 – 0.16) | -0.03 | 0.510 | -0.16  (-0.45 – 0.12) | -0.06 | 0.268 |
| PSWQ-A at baseline | -0.25  (-0.32 – -0.17) | -0.27 | **<0.001** | -0.06  (-0.13 – 0.01) | -0.06 | 0.120 | 0.04  (-0.06 – 0.13) | 0.04 | 0.441 | -0.05  (-0.15 – 0.06) | -0.04 | 0.418 |
| WEMWBS at baseline |  |  |  | 0.64  (0.56 – 0.71) | 0.60 | **<0.001** | 0.47  (0.37 – 0.57) | 0.41 | **<0.001** | 0.38  (0.27 – 0.49) | 0.32 | **<0.001** |
| Condition  (self-monitoring) |  |  |  | -0.55  (-1.67 – 0.58) | -0.07 | 0.343 | 0.63  (-0.82 – 2.08) | 0.08 | 0.393 | 0.90  (-0.81 – 2.61) | 0.11 | 0.300 |
| Condition (self-  Monitoring + EC) |  |  |  | 0.16  (-0.90 – 1.21) | 0.02 | 0.769 | -0.04  (-1.39 – 1.31) | -0.01 | 0.953 | 0.85  (-0.71 – 2.42) | 0.10 | 0.285 |
| Observations | 665 | | | 574 | | | 554 | | | 501 | | |
| R^2^ / R^2^ adjusted | 0.293 / 0.288 | | | 0.475 / 0.467 | | | 0.283 / 0.273 | | | 0.182 / 0.168 | | |

*Linear regressions testing the effects of RNT inertia and variability on mental well-being (sum score on the WEMWBS)*

*Note. B* (CI)= unstandardized regression coefficient (with 95% confidence interval), *β* = standardized regression coefficient, *p* = raw *p*-value; bold *p*-values denote significance below *α* = 0.017 (Bonferroni-corrected for multiple dependent variables); *R² (adjusted)* = (adjusted) coefficient of determination; , self-monitoring = self-monitoring only app, self-monitoring + EC = self-monitoring + personalized emotional competence training self-help via app. Reference group for condition is self-monitoring + generic cognitive-behavioral therapy self-help via app.

**Table S4**

*Linear regressions testing the effects of RNT instability on depressive symptoms (sum score on the PHQ-9)*

|  | **Baseline** | | | **One-month Follow-up** | | | **Three-month Follow-up** | | | **Twelve-month Follow-up** | | |
| --- | --- | --- | --- | --- | --- | --- | --- | --- | --- | --- | --- | --- |
| *Predictors* | *B (CI)* | *β* | *p* | *B (CI)* | *β* | *p* | *B (CI)* | *β* | *p* | *B (CI)* | *β* | *p* |
| PTQ_EMA_ (instability) | -0.12  (-0.21 – -0.03) | -0.07 | **0.008** | -0.03  (-0.14 – 0.08) | -0.01 | 0.625 | 0.06  (-0.07 – 0.19) | 0.03 | 0.370 | 0.08  (-0.07 – 0.23) | 0.04 | 0.288 |
| PTQ_EMA_ (mean) | 0.14  (0.09 – 0.18) | 0.17 | **<0.001** | 0.23  (0.18 – 0.29) | 0.26 | **<0.001** | 0.19  (0.13 – 0.26) | 0.20 | **<0.001** | 0.15  (0.07 – 0.23) | 0.14 | **<0.001** |
| RRS-B at baseline | 0.33  (0.25 – 0.40) | 0.27 | **<0.001** | 0.12  (0.02 – 0.2)1 | 0.08 | **0.014** | 0.16  (0.05 – 0.27) | 0.11 | **0.005** | 0.07  (-0.07 – 0.20) | 0.04 | 0.323 |
| PSWQ-A at baseline | 0.14  (0.11 – 0.16) | 0.29 | **<0.001** | 0.02  (-0.01 – 0.06) | 0.04 | 0.243 | -0.02  (-0.06 – 0.02) | -0.03 | 0.407 | 0.02  (-0.03 – 0.07) | 0.03 | 0.509 |
| PHQ-9 at baseline |  |  |  | 0.41  (0.33 – 0.48) | 0.35 | **<0.001** | 0.41  (0.32 – 0.50) | 0.33 | **<0.001** | 0.36  (0.25 – 0.46) | 0.28 | **<0.001** |
| Condition  (self-monitoring) |  |  |  | 0.59  (0.02 – 1.15) | 0.15 | 0.042 | 0.50  (-0.17 – 1.16) | 0.12 | 0.143 | -0.00  (-0.80 – 0.79) | -0.00 | 0.998 |
| Condition (self-  Monitoring + EC) |  |  |  | 0.30  (-0.23 – 0.84) | 0.08 | 0.269 | 0.18  (-0.45 – 0.82) | 0.04 | 0.573 | 0.16  (-0.59 – 0.91) | 0.03 | 0.678 |
| Observations | 994 | | | 820 | | | 771 | | | 698 | | |
| *R^2^* / *R^2^*adjusted | 0.329 / 0.326 | | | 0.339 / 0.333 | | | 0.253 / 0.246 | | | 0.160 / 0.152 | | |

*Note. B* (CI)= unstandardized regression coefficient (with 95% confidence interval), *β* = standardized regression coefficient, *p* = raw *p*-value; bold *p*-values denote significance below *α* = 0.017 (Bonferroni-corrected for multiple dependent variables); *R² (adjusted)* = (adjusted) coefficient of determination; , self-monitoring = self-monitoring only app, self-monitoring + EC = self-monitoring + personalized emotional competence training self-help via app. Reference group for condition is self-monitoring + generic cognitive-behavioral therapy self-help via app.

**Table S5**

|  | **Baseline** | | | **One-month Follow-up** | | | **Three-month Follow-up** | | | **Twelve-month Follow-up** | | |
| --- | --- | --- | --- | --- | --- | --- | --- | --- | --- | --- | --- | --- |
| *Predictors* | *B (CI)* | *β* | *p* | *B (CI)* | *β* | *p* | *B (CI)* | *β* | *p* | *B (CI)* | *β* | *p* |
| PTQ_EMA_ (instability) | -0.02  (-0.11 – 0.06) | -0.01 | 0.585 | 0.07  (-0.02 – 0.17) | 0.04 | 0.130 | 0.14  (0.02 – 0.27) | 0.07 | 0.021 | -0.01  (-0.15 – 0.13) | -0.00 | 0.902 |
| PTQ_EMA_ (mean) | 0.15  (0.11 – 0.19) | 0.18 | **<0.001** | 0.22  (0.17 – 0.27) | 0.25 | **<0.001** | 0.19  (0.13 – 0.26) | 0.21 | **<0.001** | 0.12  (0.05 – 0.20) | 0.13 | **0.001** |
| RRS-B at baseline | 0.25  (0.18 – 0.32) | 0.19 | **<0.001** | 0.05  (-0.03 – 0.13) | 0.04 | 0.241 | 0.10  (-0.01 – 0.21) | 0.07 | 0.064 | 0.07  (-0.05 – 0.20) | 0.05 | 0.262 |
| PSWQ-A at baseline | 0.23  (0.20 – 0.26) | 0.46 | **<0.001** | 0.11  (0.08 – 0.15) | 0.23 | **<0.001** | 0.07  (0.02 – 0.11) | 0.12 | **0.003** | 0.07  (0.02 – 0.12) | 0.12 | **0.010** |
| GAD-7 at baseline |  |  |  | -0.06  (-0.56 – 0.43) | -0.02 | 0.803 | -0.05  (-0.68 – 0.59) | -0.01 | 0.885 | -0.08  (-0.83 – 0.66) | -0.02 | 0.825 |
| Condition  (self-monitoring) |  |  |  | 0.35  (0.28 – 0.42) | 0.34 | **<0.001** | 0.29  (0.20 – 0.38) | 0.26 | **<0.001** | 0.24  (0.13 – 0.34) | 0.21 | **<0.001** |
| Condition (self-  Monitoring + EC) |  |  |  | -0.16  (-0.63 – 0.30) | -0.04 | 0.497 | -0.12  (-0.73 – 0.48) | -0.03 | 0.688 | 0.21  (-0.49 – 0.91) | 0.05 | 0.555 |
| Observations | 994 | | | 820 | | | 774 | | | 695 | | |
| *R^2^*/ *R^2^*adjusted | 0.463 / 0.460 | | | 0.477 / 0.473 | | | 0.284 / 0.277 | | | 0.170 / 0.162 | | |

*Linear regressions testing the effects of RNT instability on generalized anxiety symptoms (sum score on the GAD-7)*

*Note. B* (CI)= unstandardized regression coefficient (with 95% confidence interval), *β* = standardized regression coefficient, *p* = raw *p*-value; bold *p*-values denote significance below *α* = 0.017 (Bonferroni-corrected for multiple dependent variables); *R² (adjusted)* = (adjusted) coefficient of determination; , self-monitoring = self-monitoring only app, self-monitoring + EC = self-monitoring + personalized emotional competence training self-help via app. Reference group for condition is self-monitoring + generic cognitive-behavioral therapy self-help via app.

**Table S6**

*Linear regressions testing the effects of RNT instability on mental well-being (sum score on the WEMWBS)*

|  | **Baseline** | | | **One-month Follow-up** | | | **Three-month Follow-up** | | | **Twelve-month Follow-up** | | |
| --- | --- | --- | --- | --- | --- | --- | --- | --- | --- | --- | --- | --- |
| *Predictors* | *B (CI)* | *β* | *p* | *B (CI)* | *β* | *p* | *B (CI)* | *β* | *p* | *B (CI)* | *β* | *p* |
| PTQ_EMA_ (instability) | 0.09  (-0.09 – 0.27) | 0.03 | 0.335 | -0.09  (-0.27 – 0.10) | -0.03 | 0.346 | -0.08  (-0.31 – 0.16) | -0.02 | 0.512 | 0.04  (-0.22 – 0.31) | 0.01 | 0.749 |
| PTQ_EMA_ (mean) | -0.25  (-0.35 – -0.16) | -0.16 | **<0.001** | -0.21  (-0.30 – -0.12) | -0.13 | **<0.001** | -0.34  (-0.46 – -0.22) | -0.19 | **<0.001** | -0.20  (-0.33 – -0.06) | -0.11 | **0.005** |
| RRS-B at baseline | -0.55  (-0.71 – -0.40) | -0.23 | **<0.001** | 0.01  (-0.15 – 0.16) | 0.00 | 0.921 | -0.05  (-0.25 – 0.16) | -0.02 | 0.666 | -0.05  (-0.28 – 0.19) | -0.02 | 0.683 |
| PSWQ-A at baseline | -0.24  (-0.30 – -0.18) | -0.26 | **<0.001** | -0.02  (-0.08 – 0.04) | -0.02 | 0.472 | 0.03  (-0.04 – 0.11) | 0.03 | 0.415 | -0.03  (-0.12 – 0.06) | -0.03 | 0.493 |
| WEMWBS at baseline |  |  |  | 0.62  (0.56 – 0.69) | 0.60 | **<0.001** | 0.48  (0.40 – 0.56) | 0.43 | **<0.001** | 0.40  (0.31 – 0.49) | 0.35 | **<0.001** |
| Condition  (self-monitoring) |  |  |  | -0.78  (-1.73 – 0.17) | -0.11 | 0.108 | 0.01  (-1.21 – 1.23) | 0.00 | 0.992 | 0.14  (-1.24 – 1.53) | 0.02 | 0.838 |
| Condition (self-  Monitoring + EC) |  |  |  | 0.11  (-0.79 – 1.01) | 0.01 | 0.811 | -0.24  (-1.40 – 0.93) | -0.03 | 0.692 | -0.17  (-1.48 – 1.14) | -0.02 | 0.799 |
| Observations | 994 | | | 823 | | | 775 | | | 698 | | |
| *R^2^* / *R^2^*adjusted | 0.261 / 0.258 | | | 0.448 / 0.443 | | | 0.270 / 0.263 | | | 0.181 / 0.173 | | |

*Note. B* (CI)= unstandardized regression coefficient (with 95% confidence interval), *β* = standardized regression coefficient, *p* = raw *p*-value; bold *p*-values denote significance below *α* = 0.017 (Bonferroni-corrected for multiple dependent variables); *R² (adjusted)* = (adjusted) coefficient of determination; , self-monitoring = self-monitoring only app, self-monitoring + EC = self-monitoring + personalized emotional competence training self-help via app. Reference group for condition is self-monitoring + generic cognitive-behavioral therapy self-help via app.
